# Complete Sequence, Analysis and Organization of the *Orgyia leucostigma* Nucleopolyhedrovirus Genome

**DOI:** 10.3390/v3112301

**Published:** 2011-11-15

**Authors:** David K. Thumbi, Robert J. M. Eveleigh, Christopher J. Lucarotti, Renée Lapointe, Robert I. Graham, Lillian Pavlik, Hilary A. M. Lauzon, Basil M. Arif

**Affiliations:** 1 Sylvar Technologies Inc., P.O. Box 636 Station A, Fredericton, New Brunswick, E3B 5A6, Canada; E-Mails: dtumbi@sylvar.ca (D.K.T.); robert.eveleigh@dal.ca (R.J.M.E); rlapointe@sylvar.ca (R.L.); 2 Natural Resources Canada, Atlantic Forestry Centre, Canadian Forest Service, 1350 Regent Street, Fredericton, New Brunswick, E3C 2G6, Canada; 3 Lancaster Environment Centre, Lancaster University, Lancaster, Lancashire, LA1 4YQ, UK; E-Mails: r.graham@lancaster.ac.uk; 4 Natural Resources Canada, Great Lakes Forestry Centre, Canadian Forest Service, 1219 Queen Street East, Sault Ste. Marie, Ontario, P6A 2E5, Canada; E-Mails: lillian.pavlik@nrcan-rncan.gc.ca (L.P); hlauzon@nrcan.gc.ca (H.A.M.L.); barif@nrcan.gc.ca (B.M.A.)

**Keywords:** alphabaculovirus, baculovirus, white-marked tussock moth

## Abstract

The complete genome of the *Orgyia leucostigma* nucleopolyhedrovirus (OrleNPV) isolated from the whitemarked tussock moth (*Orgyia leucostigma*, Lymantridae: Lepidoptera) was sequenced, analyzed, and compared to other baculovirus genomes. The size of the OrleNPV genome was 156,179 base pairs (bp) and had a G+C content of 39%. The genome encoded 135 putative open reading frames (ORFs), which occupied 79% of the entire genome sequence. Three inhibitor of apoptosis (ORFs 16, 43 and 63), and five baculovirus repeated ORFs (*bro-a* through *bro-e*) were interspersed in the OrleNPV genome. In addition to six direct repeat (*drs*), a common feature shared among most baculoviruses, OrleNPV genome contained three homologous regions (*hrs*) that are located in the latter half of the genome. The presence of an F-protein homologue and the results from phylogenetic analyses placed OrleNPV in the genus *Alphabaculovirus*, group II. Overall, OrleNPV appears to be most closely related to group II alphabaculoviruses *Ectropis obliqua* (EcobNPV), *Apocheima cinerarium* (ApciNPV), *Euproctis pseudoconspersa* (EupsNPV), and *Clanis bilineata* (ClbiNPV).

## 1. Introduction

The family *Baculoviridae* consists of rod-shaped, enveloped, and occluded viruses mainly pathogenic to insects in the orders Lepidoptera, Diptera and Hymenoptera [1]. In recent years, there has been continued interest in baculovirus genome studies mainly due to their application as biopesticides of major insect pests in forests and agriculture [2–4], and also as vectors for protein expression and gene therapy [5–6].

Baculoviruses genomes are characterized by their large, covalently-closed, circular, double-stranded DNA molecules containing numerous open reading frames (ORFs) which are distributed on both strands. Baculoviruses are classified into four genera based on host specificities and phylogenetic analysis; *Alphabaculovirus* (lepidopteran-specific nucleopolyhedroviruses or NPVs), *Betabaculovirus* (lepidopteran-specific granuloviruses or GVs), *Gammabaculovirus* (hymenopteran-specific NPVs), and *Deltabaculovirus* (dipteran-specific NPVs) [7]. Alphabaculoviruses are subdivided further, based, in part, on the presence of genes encoding different glycoproteins that serve similar functions associated with budded virions (BV), *gp64* in group I and *f-protein* in group II [8–10]. Baculovirus genomes range in size from 81,755 bp for *Neodiprion lecontei* NPV (NeleNPV) to 178,733 bp for *Xestia c-nigrum* GV (XecnGV) [11,12]. To date, 58 baculovirus genomes including 41 *Alphabaculovirus*, 13 *Betabaculovirus*, three *Gammabaculovirus* and one *Deltabaculovirus* have been sequenced and are available in the National Centre for Biotechnology Information (NCBI) database (Table 1).

Baculoviruses exhibit two phenotypically distinct phenotypes: (i) budded virions (BV), and (ii) occlusion derived virions (ODV) that are produced during the early and late phases of virus replication, respectively. The ODV phenotypes for granuloviruses (GV) (*Betabaculovirus*) are embedded in a small granulin protein matrix unlike those of nucleopolyhedroviruses (NPV) that are contained in a large polyhedrin matrix. The OBs of GVs contain single virions while NPVs contain multiple virions within the polyhedrin matrix.

The ODVs of the alphabaculovirus infecting the whitemarked tussock moth (WMTM) *Orgyia leucostigma* (L.) have single nucleocapsids per virion. The WMTM is a common pest of balsam fir trees (*Abies balsamea* (L.) Miller) in Canada but will feed on a number of conifer, decidiuous trees, and agricultural crops such as blueberries (*Vaccinium* spp.). OrleNPV has been documented to contribute to the collapse of WMTM populations in Atlantic Canada [13]. Experimental applications of OrleNPV for the control of WMTM infestations have been made in Nova Scotia [14]. To better understand the molecular basis of OrleNPV pathogenicity, viral DNA from a Nova Scotia isolate was purified, sequenced and compared with other baculoviruses using various phylogenetic tools. The OrleNPV genome was found to be 156,179 bp in size and was confirmed to belong in the genus *Alphabaculovirus*, group II.

## 2. Results and Discussion

### 2.1. Nucleotide Sequence Analysis

**Figure 1.**
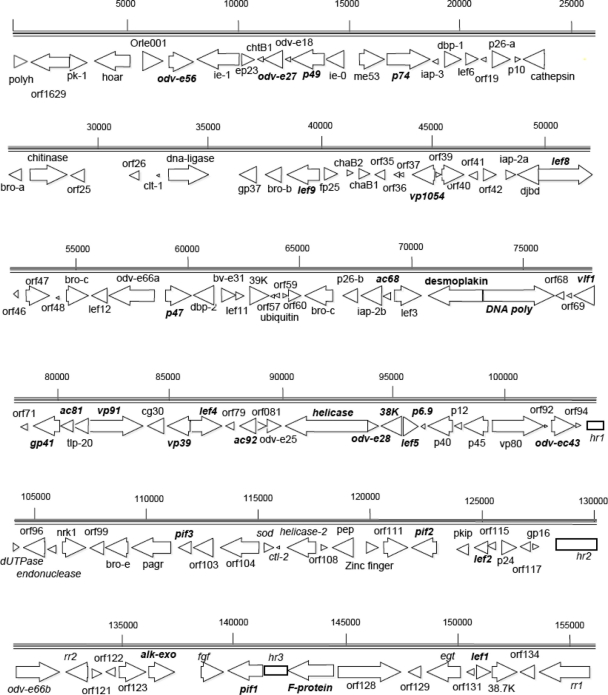
Linear map of *Orgyia leucostigma* nucleopolyhedrovirus (OrleNPV) genome. The arrows and arrow heads indicate the orientation of the predicted open reading frames (ORFs). The rectangles indicate homologous regions (*hrs*). The number above the horizontal lines denotes the nucleotide position (bp) relative to the start codon of polyhedrin ORF. Thirty-one baculovirus core genes are shown in bold letters.

The circular OrleNPV genome was 156,179 bp in size, making it the eighth largest baculovirus genome sequenced to date, with others ranging from 81,755 bp for *Neodiprion lecontei* NPV (NeleNPV) [12] to 178,733 bp for *Xestia c-nigrum* GV (XecnGV) [15]. The OrleNPV genome is AT-rich having only 39% G+Cs, lower than the average GC content of group I (44.9 %) and group II (41.6%) alphabaculoviruses [16]. As originally defined, [17], the adenine of methionine start codon of the polyhedrin gene represented the zero point on the OrleNPV physical map (Figure 1), and was designated as ORF 1 (Table 2). Overall, 135 putative ORFs, with a minimal size of 50 amino acids and with promoter motifs corresponding to various transcriptional profiles, were detected and encoded for 79% of the total genome (Tables 1 and 2). Fifty-eight (42.9%) of the ORFs were forward oriented, whereas 77 (57.1%) were reverse oriented (Figure 1). Interestingly, OrleNPV encodes for relatively fewer genes (135) compared to other baculoviruses where the average number of ORFs is 140.5. This could be attributed to the presence of several copies of *hrs*, *drs*, and large intergenic regions, which collectively account for 21 % on the entire genome. Multiple copies of *hrs* and *drs* may be a replication strategy for the virus to initiate DNA replication from several regions leading to rapid rate of DNA replication in the host system.

### 2.2. Gene Homology

The OrleNPV genome was compared to *Autographa californica* MNPV (AcMNPV), *Chrysodeixis chalcites* NPV (ChchNPV), *Ectropis obliqua* NPV (EcobNPV), and *Orgyia pseudotsugata* MNPV (OpMNPV) genomes (Table 2). All 62 conserved alphabaculovirus genes, including 31 core genes found in all baculoviruses [12,16,18], were clearly identified in OrleNPV. Of the 135 OrleNPV putative ORFs, 134 were homologous to other baculovirus ORFs and one ORF (ORF5, *Orle001*) was unique to OrleNPV (Table 3). OrleNPV genome shared the largest number of homologues with group II alphabaculoviruses: 114 (84%) with EcobNPV and 103 (76%) with ChchNPV. The number of homologues in common between OrleNPV and group I alphabaculoviruses, AcMNPV and OpMNPV were 87 (64%) and 81 (60%), respectively. The mean amino acid identity between OrleNPV and group II alphabaculoviruses was EcobNPV 52.4% ± 16.6 standard deviation, and ChchNPV 48.2% ± 15.1. The highest mean amino acid identity between OrleNPV and a group I alphabaculovirus was with AcMNPV at 43.1% ± 14.7. These results support OrleNPV as a group II alphabaculovirus with further evidence provided by the presence of the group II specific *f-protein* gene and absence of the group I specific *gp64* [9,10,18,19]. *Polyhedrin* was the most conserved ORF between OrleNPV and other alphabaculoviruses with highest shared putative amino acid identities to EcobNPV (96%), ChchNPV (95%), AcMNPV (89%), and OpMNPV (86%) (Table 2). Very late expression factor 1 (*vlf-1*, ORF70) was the second most conserved ORFs between OrleNPV and other alphabaculoviruses; EcobNPV (84%), ChchNPV (75%), AcMNPV (74%), and OpMNPV (70%).

### 2.3. Gene Organization

**Figure 2.**
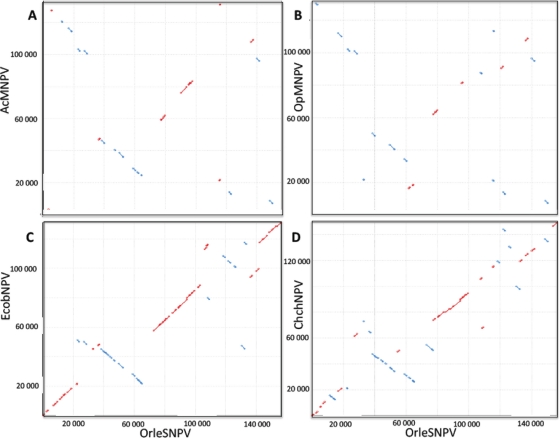
Gene-parity plot analysis. Gene parity comparison of gene order of OrleNPV with (**A**) *Autographa californica* MNPV (AcMNPV), (**B**) *Orgyia pseudotsugata* MNPV (OpMNPV), (**C**) *Ectropis obliqua* (EcobNPV), and (**D**) *Chrysodeixis chalcites* NPV (ChchNPV). Blue dots indicate inversions in relation to the OrleNPV gene order. Numbers on the x and y axes are nucleotide position (bp) relative to the polyhedrin start codon.

Gene parity plots were used to reveal the gene order between two baculoviruses, where closely related baculoviruses show colinear arrangement of genes and, conversely, colinearity decreases with increased divergence between baculoviruses [12,20]. Comparisons of relative gene order between OrleNPV and EcobNPV, ChchNPV, AcMNPV or OpMNPV homologues revealed five gene clusters that were conserved in these six alphabaculoviruses (Figure 2). The gene clusters consisted of 57 genes where 22 (76%) core baculovirus genes and 23 (72%) lepidopteran specific genes were identified. Gene cluster 5 showed the largest collinear segment which contained a core cluster of four genes, *helicase*, *ac96 (odv-e28)*, *38K* and *lef5*, that are thought to be present in all baculoviruses [18]. The gene arrangements of these five gene clusters indicate further that OrleNPV is more closely related to group II than group I alphabaculoviruses. Inversions in gene clusters 3 and 4 were identified in all six of these group I alphabaculovirues (Figure 2) and ChchNPV, a group II alphabaculovirus, showed inversion orientations in gene clusters 2 and 5 similar to group I alphabaculoviruses, AcMNPV and OpMNPV. These results are in agreement with a recent study, which showed similar gene arrangements between ClbiNPV and OrleNPV [21].

### 2.4. Homologous Regions and Direct Repeats

**Figure 3.**
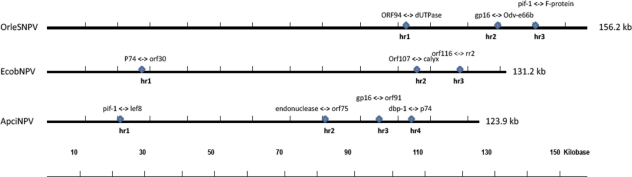
Comparisons of *hrs* distribution. Linear map showing relative genomic locations of *hrs* (blue diamond shapes) of closely related baculoviruses; OrleNPV, EcobNPV and ApciNPV. ORFs flanking each of the *hrs* are indicated above each *hr*.

**Figure 4.**
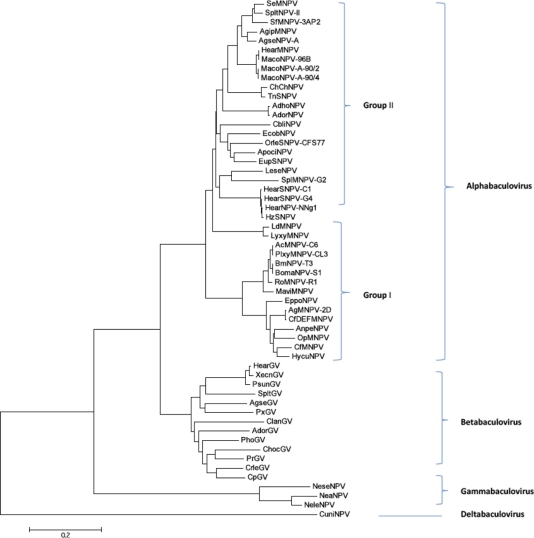
Baculovirus Phylogeny. The phylogenetic tree was generated from concatenated amino acid sequences alignment of *lef-8* and *pif-2* genes of 56 of the 58 currently sequenced genomes. Evolutionary analyses were conducted in using MEGA5 software.

Homologous regions (*hrs*) are *cis*-acting elements present in many baculovirus genomes and most are characterized by the presence of several copies of direct repeats and imperfect palindromes. Comparisons of 57 sequenced baculovirus genomes showed that 47 contain *hrs* and eight, including *Trichoplusia ni* SNPV (TnSNPV), *Adoxophyles orona* GV (AdorGV), *Agrotis segetum* GV (AgseGV), *Cydia pomonella* GV (CpGV), *Crytophlebia leucotreta* GV (CrleGV), *Pieris rapae* GV (PrGV), SlGV, and NeleNPV have no *hrs* (Table 1). *Spodoptera litura* SMNPV genome (SpltMNPV-G2), contains 17 *hrs*, which is the highest number of *hrs* reported to date [22]. Although the *in vivo* origin(s) of baculovirus DNA replication (*oris*) remain unclear, *hrs* have been implicated as putative *oris*, and as enhancers of early gene expression using transient replication assays [23–25]. In addition, the interpalindromic sequences of baculovirus *hrs* contain certain cAMP response elements (CRE), which could bind host transcriptions factors of the bZIP family and possibly provide a synergy for the binding of IE-1 to the *hrs* [26,27]. In other baculovirus genera, particularly some betabaculoviruses, *hrs* exhibit both spatial and sequence heterogeneity and are devoid of the consensus palidromic sequences such as those found in the type baculovirus AcMNPV. For example, of the 9 *hrs* of XecnGV, *hrs* 1–8 contains about 120 bp long imperfect 3–6 direct repeats with no obvious palindromes and are interspersed within AT-rich regions, but the other *hr* is located within an ORF [15]. The OrleNPV genome contained three *hrs* that were located in the latter half of the genome (Table 2). Like other alphabaculoviruses, OrleNPV *hrs* were characterized by the presence of a single tandemly repeated imperfect palindrome sequence within a direct repeat of two to three copies. In AcMNPV genome, however, *hrs* have common repetitive elements with an imperfect palindrome of about 30 bp and are interspersed at multiple loci in the genome [27]. The size of the three OrleNPV *hrs* was variable (*hr*1 305 bp, *hr*2 219 bp and *hr*3 324 bp, (Table 2). Similar variations were observed in EcobNPV, which appears to be closely related to OrleNPV [28]. However, given the different numbers of *hrs*, the combined size of the OrleNPV *hrs* is relatively smaller (848 bp or 0.5% of the genome) compared to the combined size of the EcobNPV *hrs* (3541 bp or 2.7% of the genome). Nevertheless, the similarity in number and arrangement of the *hrs* (Figure 3), coupled with the phylogenetic analysis (Figure 4) strongly support the possibility of the two viruses sharing a common ancestor. In both OrleNPV and EcobNPV, there are indications of genome variability and arrangements around *hr*3. Here, two copies of OrleNPV ribonucleotide reductase genes (*rr1* and *rr2*) are located on either side of the *hr*3 and in the same orientation. In EcobNPV genome, however, both *rr1* and *rr2* genes appear on the right side of *hr3* and in opposite orientations. Moreover, EcobNPV *pif1* gene does not flank *hr*3 as is the case of OrleNPV genome. Together, these findings suggest that, although both viruses appear to share a common ancestor (Figure 4), gene rearrangements around *hr*3 could have occurred through homologous recombination as the two viruses adapted in their specific hosts. Similar observations have been reported in other alphabaculoviruses although at different *hr* loci [29–31].

In addition to *hrs*, the OrleNPV genome contained six direct AT-rich repeat regions (*drs*) (except rep 5) containing 2 to 10 copies of direct tandem repeat sequences, ranging in size from 31 to 97 bp (Table 2). Common in other baculoviruses, *drs* have been implicated as origins of DNA replication [11,32,33]. They have also been shown to be more complex in structure compared to *hrs* and have similar sequence organization to those of eukaryotic *oris* [34–37]. Interestingly, the six *drs* located in the first half of the OrleNPV genome are absent in EcobNPV, ApciNPV, and *Ectropis pseudoconspersa* NPV (EupsNPV) in spite of their evolutionary relatedness. This observation suggests that these viruses may utilize different *in vivo* origins of DNA replication. This notion is supported by recent findings, which showed that none of the *hrs* is essential for baculovirus DNA replication [38]. Although structurally distinct to those of OrleNPV, non-*hr* repeats, have been reported in OpMNPV [29]. Since non-*hrs* mimics eukaryotic *oris*, and that OrleNPV and OpMNPV infect the same hosts, it is likely that the non-*hrs* were acquired from the host and may play an important role in viral DNA replication. It is worth noting that the only gene unique to OrleNPV (*orle001*) is flanked by *rep1* and *rep2* (Table 2). This gene may have been acquired from insect host and thus the *drs* may play a role in the horizontal transfer of genes into viral genomes. Although the molecular function of *orle001* is unknown, based on its close proximity to these non*-hr oris*, it is also possible that *orle001* product interacts with other replication machinery at the *drs cis*-acting elements during viral gene expression or DNA replication.

### 2.5. Baculovirus-Repeated ORFs

*Bro* genes are common in many baculovirus genomes and constitute a family of repetitive genes which vary in number and distribution. Among the current, sequenced baculovirus genomes, LdMNPV has the highest number of *bro* genes (16 copies), but they are absent in 13 baculovirus genomes including *Maruca vitrata* NPV (MaviNPV), *Rachiplusia ou* MNPV (RoMNPV), ApciNPV, *Spodoptera exigua* MNPV (SeMNPV), AdorGV, AgseGV, (ChocGV), CrleGV, PrGV, *Plutella xylostella* GV (PxGV), *Neodiprion abietis* NPV (NeabNPV), NeleNPV, and *Neodiprion sertifer* NPV (NeseNPV) (Table 1). In addition to baculoviruses, homologues of *bro* genes have been reported in ascoviruses, iridoviruses, entomopoxviruses, and class II transposons of prokaryotes [39]. Although their role during baculovirus replication is not clear, *bro* gene products have been implicated as potential DNA binding protein involved in host transcriptional regulation and DNA replication [40,41]. Other putative functions of *bro* genes, which may depend on the target insect host, include enhancement of late phase of virus replication [39] and as CRM1-dependent nuclear export shuttle proteins [42]. Five *bro* genes were identified in OrleNPV (ORFs 23, 30, 49, 61 and 100) and were designated as *bro-a*, *bro-b*, *bro-c*, *bro-d*, and *bro-e* based on their order of appearance in the genome (Table 2). *Bro-a* shares 27% and 38% identity with its respective homologues in ChchNPV and LdMNPV. *Bro-b* shares 25% identity with its LdMNPV homologue. *Bro-c* shares between 21% to 56% identity with its homologues in AcMNPV, EcobNPV, ChchNPV, and LdMNPV. *Bro-d* shares between 25% to 28% identity with its homologues in AcMNPV, EcobNPV, and LdMNPV. *Bro-e* shares between 47% to 64% identity with its homologues in EcobNPV, ChchNPV and LdMNPV. Based on LdMNPV Bro protein classification, *bro-d* belongs to group I, *bro-b*, *bro-c,* and *bro-e* (where *bro-c* and *bro-e* are more similar to each other than *bro-b*) belong to group II, and *bro-a* belongs to group III [43]. As shown in the phylogenic tree (Figure 4), ApciNPV is phylogenetically related to OrleNPV but lacks *bro* genes suggesting that the former may have lost *bro* genes by recombination as the viruses specialized to their hosts. Phylogenetic analysis of a multigene family of *bro-like* genes of other invertebrate DNA viruses and bacteria suggested that *bro* genes resulted from recombination events leading to loss, duplication, and acquisition of genes by horizontal gene transfer [39].

### 2.6. Genes with Two Homologues

Three genes, *odv-e66*, *p26*, and *dbp,* have two copies interspersed within the OrleNPV genome. A homologue of *odv-e66* (*ac46*) has been shown to contain an inner nuclear membrane sorting motif at the N-terminal 33 aa, which promotes selective intracellular trafficking of proteins to the inner nuclear membrane [44]. Two copies of *odv-e66* are also present in four other alphabaculoviruses including; EcobNPV (Ecob31 and Ecob48), SeMNPV (Se57 and Se114), *Mamestra configurata* NPV-A (MacoNPV-A) (ORFs 78 and 114), and MacoNPV-B (ORFs 77 and 143) [28,45–47]. Like OrleNPV, two copies of auxiliary gene *p26* [ORFs 20 (*p26a*) and 62 (*p26b*)] can be found in other group II alphabaculoviruses including SeMNPV, AgseNPV-A, MacoNPV-B, ChchNPV, and TnSNPV and it has also been shown to be present in the group I alphabaculovirus, CfMNPV (Cf7 and Cf128) [30]. The placement of *p26a* (ORF 20) between Ac29 (ORF19) and p10 gene regions in OrleNPV was conserved as its homologues in EcobNPV (Ecob18), SeMNPV (Se129), AgseNPV-A (Asnv142), MacoNPV-B (MacoB157), ChchNPV (Chch19) and TnSNPV (Tn19) are also located between these two ORFs. However, the location of *p26b* (ORF62) homologues was more divergent in these same baculoviruses. OrleNPV is the fifth baculovirus identified to contain two copies of DNA-binding protein [*dbp-1* (ORF 17), *dbp-2* (ORF53)], the others being EcobNPV [28], LdMNPV [43], ClbiNPV [21], and *Lymantria xylina* MNPV (LyxyMNPV) [48]. A homologue of this gene in AcMNPV (*ac25*) has been implicated as encoding a single stranded DNA binding protein required for synthesis of normal levels of viral DNA, formation of virogenic stroma, and processing of replication intermediates by binding nascent DNA molecules [49,50]. OrleNPV *dbp-1* shares low identities with Ecob27 (23 %) and Ld37 (25%) *dbp-1* and OrleNPV *dbp-2* shares slight higher identities with Ecob15 (27%) (Table 2). DBP-2 (ORF53) shared high aa identity with EupsNPV GP044 (47 %). Although the significance of having two copies of the above mentioned three genes in OrleNPV and other alphabaculoviruses is unclear, it is possible that one copy may act as a genetic backup to compensate the function in case either of the copy becomes dysfunctional due to mutations. Alternatively, gene duplication may contribute to unconstrained evolution, thus, possible adaptation to new environments (hosts).

### 2.7. Inhibitors of Apoptosis (iap)

Baculovirus inhibitors of apoptosis (*iap*) genes help to circumvent insect defense mechanisms involving programmed death of virus-infected cells and may act as host range factors [51]. Some alphabaculoviruses such as AcMNPV, RoMNPV, *Bombyx mori* NPV (BmNPV), *Maruca vitrata* NPV (MaviNPV), and SpltMNPV contain the *p35* family of anti-apoptosis genes in addition to the *iap* family of genes. Both families of anti-apoptotic genes have been shown to have complementary functions, for example, replication of AcMNPV *p35* mutant was rescued by *iap* genes from other baculoviruses [52,53]. OrleNPV genome contained three *iap* genes but lacks *p35*. ORF 16 (*iap3*) showed highest identity with group I alphabaculovirus, *Choristoneura fumiferana* MNPV (CfMNPV) *iap3* (61%) and with group II, TnSNPV *iap1* (54%). ORF 43 (*iap2*), however, showed highest identity to EupsNPV *iap1* (35%) and *Anticarsia gemmatalis* MNPV (AgMNPV-2D) *iap-3* (30%) in respective group II and I alphabaculoviruses. The other copy of OrleNPV *iap2* (ORF 63) showed highest identities with *iap2* of group II alphabaculoviruses, EcobNPV (33%) and ChchNPV (32%) (Table 2). All three proteins contained one copy of baculovirus inhibitor of apoptosis repeats (BIRs). BIRs contain a zinger-finger domain and a RING domain at the C-terminus and are involved in binding apoptosis-inducing proteins [54,55]. ORF 43 and 63 contained a zinc finger at the C-terminus, whereas ORF16 lacked a zinc finger domain which suggests that they may be involved in binding different forms of host apoptosis proteins.

### 2.8. Auxiliary Genes

Baculovirus auxillary genes are not essential for viral gene expression, DNA replication or morphogenesis, but provide a selective advantage for virus survival [19]. OrleNPV genome contained 11 auxiliary genes including; alkaline exonuclease (*alk-exo*), *chitinase*, cathepsin L-like proteinase (*v-cath*), ecdysteroid UDP glycosyltransferase (*egt*), fibroblast growth factor (*fgf*), *p10*, protein kinase-1 (*pk-1*), superoxide dismutase (*sod*), ubiquitin (*ubi*), conotoxin-like protein 1 and 2 (*ctl-1 and ctl-2*). Most of these genes exhibited 45–83% putative amino acid identities to their respective homologues in EcobNPV [28] (Table 2). OrleNPV genome encodes two copies of *ctl* genes as do *Hyphantria cunea* NPV (HycuNPV), OpMNPV and LdMNPV genomes. OrleNPV *ctl-1* shares 86% putative amino acid identity with its ChchNPV homologue [57] (Table 2). Auxiliary genes not found in OrleNPV were actin rearrangement-inducing factor-1 (*arif-1*), proliferating nuclear antigen (*pcna*), viral enhancing factor-1 (*vef-1*) and *ptp-1*.

### 2.9. Phylogenetic Analysis

To date, 31 core genes have been used to elucidate the evolutionary relationships of various baculovirus species [16]. In this study, the phylogenetic tree was generated based on the concatemers of ac22 homologue (*pif-2*) and *lef-8* genes. These two genes were previously shown to generate robust tree that is comparable to that of baculovirus core genes [58] from the current baculovirus genomes available in the NCBI database. The tree showed a clear separation of baculoviruses into their current scheme of classification (Figure 4) [7]. In addition, group I alphabaculoviruses and betabaculoviruses showed clear separation into two distinct clades (a and b), which have been reported in previous studies using the analysis of all baculovirus core genes [7,16,58]. This information further highlights the significance of these two conserved genes, albeit non-essential for replication, in elucidating the evolution of baculoviruses in their respective hosts [58]. Our results indicate that OrleNPV is most closely related to EcobNPV, ApciNPV, EupsNPV, and ClbiNPV (Figure 4).

## 3. Experimental Section

### 3.1. Virus Propagation, DNA Extraction and Purification

Third-instar *O. leucostigma* larvae were fed OrleNPV at a rate of 5 × 10^4^ OBs per larva. Infected larvae were reared on artificial diet and were monitored for viral pathogenesis at various days post infection (dpi). Occlusion bodies were isolated from infected larvae as previously described with minor modifications [59,60]. In brief, 20 larvae were collected 10 dpi, homogenized with a hand blender, and stirred for 2 h in 0.5% SDS. The homogenate was filtered through cheese cloth and centrifuged at 7000 × g for 15 min at 15 °C. The pellet containing the OBs and insect debris was washed three times in deionized water by centrifugation as describe above. The final pellet of purified OBs was resuspended in 5 mL of sterile distilled water. OBs were dissolved in an alkaline solution (1.0 M sodium carbonate and 0.4 M sodium thioglycolate) to release the occlusion derived virus (ODV). The solution was centrifuged at 1000 × g to remove debris and undissolved OBs. ODV was purified in a continuous 10–45% sucrose gradient as previously described [61]. The band containing the ODV was collected, diluted with TE (10mM Tris and 1.0 mM EDTA, pH 8.0), and centrifuged at 22000 rpm (SW28 rotor Beckman ultracentrifuge) for 2 h at 4 °C. The pellet was suspended in 500 μL of TE and digested with proteinase K (25 μL, 20 mg/mL) along with 1% N-lauryl-sarcosine (final concentration) for 2 h at 37 °C. Viral DNA was further purified by a CsCl gradient approach as previously described [60] followed by dialysis of viral DNA against several changes of TE for 72 h at 4 °C. Purified DNA was resuspended in TE, and quantified spectrophometrically to ascertain DNA yield and purity.

### 3.2. DNA Sequencing and Analysis

OrleNPV genome was determined using the shotgun sequence approach as previously described [12]. In brief, total genomic DNA was sheared into small random fragments by nebulization and cloned to generate an overlapping genomic library. Purified template DNA from the genomic library was sequenced in an ABI Prisms® 3700 analyzer using the BigDye® terminator chemistry (v.3.0/3) as per Agencourt Bioscience Corporation (Beverly, MA, USA) sequencing specifications. The overall sequence obtained accounted for a 12-fold genomic coverage. The OrleNPV genome was assembled by Agencourt BioScience. Lasergene DNAStar SeqManPro (version 7.2) was used to manually edit and verify the contiguous sequence data. Genomic data was remotely submitted to Emboss software suite and start-to-stop translational searching was performed using getOrf program [62]. Open reading frames (ORFs) encoding 50 or more amino acids with minimal overlap were accepted as putative genes based on the established criteria [17]. NCBI web blast perl script was used to submit relevant ORFs to the GenBank. Homologue identification was done using standard protein-protein BLAST (blastp) with default settings [63].

Sequences 160 bp upstream of the predicted ORF start codons were analyzed for potential promoter motifs using WebLogo [64]. Upstream sequences were also scanned for exact matches using regular expression notation native to perl for commonly known baculovirus promoter elements, including the TATA sequence (TATA/TATAW), CAKT and DTAAG, within 160 bp or 40 to 20 bp regions. Further motif optimizations were preformed on BioProspector and AlignACE results using BioOptimizer v 3.0 [65]. Repeat regions including *hrs* and direct repeat regions were identified using Tandem repeat finder [66], Emboss Palindrome [62], and Reputer [67].

### 3.3. Gene homology and Phylogenetic Analysis

Predicted ORFs were compared with homologues in four alphabaculoviruses, which included AcMNPV; NCBI reference (NC_001623) MNPV OpMNPV (NC_001875), ChchNPV (NC_007151), EcobNPV (NC_008586). Gene parity plots were used to analyze the gene order of OrleNPV relative to those representatives of group I and group II alphabaculoviruses mentioned above. Phylogenetic tree was generated using concatenated amino acid sequences derived from *lef-8* and *pif-2* genes for the 56 complete baculovirus genomes that were available in the NCBI database at the time of analysis. The tree was inferred using neighbour-joining method using the MEGA5 program [68].

## 4. Conclusions

OrleNPV genome is the eighth largest baculovirus genome sequenced to date (157, 179 bp) and encodes for a total of 135 ORFs with one being unique to the virus. Interspersed within the genome are three *hrs*, five *bros*, and three *iap* genes, which are common features in most baculovirus genomes. Duplicate copies of *dbp*, *odv-e66*, and *p26* were present in OrleNPV genome. Based on phylogenetic analysis and gene arrangements, OrleNPV appears to be most closely related to the group II alphabaculoviruses EcobNPV, ApciNPV, EupsNPV, and ClbiNPV. Together, the OrleNPV genomic data would be helpful components for future analysis of emerging baculovirus genomes and also in providing a deeper understanding of the molecular basis of OrleNPV pathogenicity.

## Figures and Tables

**Table 1.** Characteristics of baculovirus genomes. The 58 currently sequenced baculovirus genomes are summarized according to their current classification scheme and alphabetical order.

**Genus (Group)**	**Virus name**	**Abbreviation**	**Length (bp)**	**G+C (%)**	**No. of ORFs**	**^1^ No. of *hrs***	**^2^ No. of *bro***	**GenBank accession**	**^3^ Reference**
*Alphabaculovirus* (Group I)	*Antheraea pernyi* NPV	AnpeNPV	126,629	53	147	3	2	DQ486030	[69]
	*Antheraea pernyi* MNPV-L2	AnpeMNPV-L2	126,246	53	145	6	2	EF207986	[70]
	*Anticarsia gemmatalis* MNPV-2D	AgMNPV-2D	132,239	44	152	9	8	DQ813662	[71]
	*Autographa californica* MNPV-C6	AcMNPV-C6	133,894	40	156	9	1	L22858	[17]
	*Bombyx mori* NPV-T3	BmNPV-T3	128,413	40	143	7	5	L33180	[72]
	*Bombyx mandarina* NPV-S1	BomaNPV-S1	126,770	40	141	7	3	FJ882854	[73]
	*Choristoneura fumiferana* MNPV	CfMNPV	129,593	50	146	5	1	AF512031	[30]
	*Choristoneura fumiferana* DEF MNPV	CfDEFMNPV	131,160	45	149	13	2	AY327402	[74]
	*Epiphyas postivittana* NPV	EppoNPV	118,584	40	136	5	1	AY043265	[75]
	*Hyphantria cunea* NPV	HycuNPV	132,959	45	148	6	5	AP009046	[76]
	*Maruca vitrata* NPV	MaviNPV	111,953	39	126	5	0	EF125867	[77]
	*Orgyia pseudotsugata* MNPV	OpMNPV	131,990	55	152	5	3	U75930	[29]
	*Plutella xylostella* MNPV (CL3)	PlxyMNPV	134,417	40	153	9	2	DQ457003	[78]
	*Rachiplusia ou* MNPV-R1	RoMNPV-R1	131,526	39	149	9	0	AY145471	[79]
*Alphabaculovirus* (Group II)	*Adoxophyes honmai* NPV	AdhoNPV	113,220	35	125	4	4	AP006270	[80]
	*Adoxophyes orana* NPV	AdorNPV	111,724	35	121	4	3	EU591746	[81]
	*Agrotis ipsilon* MNPV	AgipMNPV	155,122	48	163	7	5	EU839994	[82]
	*Agrotis segetum* NPV-A	AgseNPV-A	147,544	45	153	5	4	DQ123841	[83]
	*Apocheima cinerarium* NPV	ApciNPV	123,876	45	118	4	0	FJ914221	NP
	*Chrysodeixis chalcites* NPV	ChchNPV	149,622	39	151	0	4	AY864330	[57]
	*Clanis bilineata* NPV	ClbiNPV	135,454	37	139	0	3	DQ504428	[21]
	*Ectropis obliqua* NPV	EcobNPV	131,204	37	126	3	2	DQ837165	[28]
	*Euproctis pseudoconspersa* NPV	EupsNPV	141,291	40	139	4	2	FJ227128	[84]
	*Helicoverpa armigera* SNPV-G4	HearSNPV-G4	131,405	39	135	5	3	AF271059	[85]
	*Helicoverpa armigera* SNPV-C1	HearSNPV-C1	130,759	38	137	5	3	AF30304	[86]
	*Helicoverpa armigera* MNPV	HearMNPV	154,196	40	162	4	6	EU730893	NP
	*Helicoverpa armigera* SNPV NNg1	HearSNPV-NNg1	132,425	39	143	5	4	AP010907	[87]
	*Helicoverpa zea* SNPV	HzSNPV	130,869	39	139	5	2	AF334030	[88]
	*Leucania separata* NPV	LeseNPV	168,041	48	169	8	10	AY394490	[89]
	*Lymantria dispar* MNPV	LdMNPV	161,046	57	164	13	16	AF081810	[43]
	*Lymantria xylina* MNPV	LyxyMNPV	156,344	53	157	13	14	GQ202541	[48]
	*Mamestra configurata* NPV-A *(90/2)*	MacoNPV-A (90/2)	155,060	41	169	4	8	U59461	[46]
	*Mamestra configurata* NPV-A (90/4)	MacoNPV-A (90/4)	153,656		168	4	7	AF539999	[90]
	*Mamestra configurata* NPV-96B	MacoNPV-B	158,482	40	169	4	7	AYI26275	[47]
	*Orgyia leucostigma* NPV	OrleNPV	156,179	39	135	3	5	EU309041	TP
	*Spodoptera exigua* MNPV	SeMNPV	135,611	43	139	6	0	AF169823	[45]
	*Spodoptera frugiperda* MNPV-3AP2	SfMNPV-3AP2	131,330	40	143	8	1	EF035042	[91]
	*Spodoptera frugiperda* MNPV-19	SfMNPV-19	132,565	40	141	8	1	EU258200	[92]
	*Spodoptera litura* MNPV-G2	SpltMNPV-G2	139,342	42	141	17	2	AF325155	[22]
	*Spodoptera litura* MNPV- II	SpltMNPV- II	148,634	44	147	7	2	EU780426	NP
	*Trichoplusia ni* SNPV	TnSNPV	134,394	39	144	0	2	DQ017380	[93]
*Betabaculovirus*	*Adoxophyles orona* GV	AdorGV	99,657	34	119	0	0	AF547984	[94]
	*Agrotis segetum* GV	AgseGV	131,680	37	132	0	0	AY522332	NP
	*Choristoneura occidentalis* GV	ChocGV	104,710	32	116	5	0	DQ333351	[61]
	*Clostera anachoreta* GV	ClanGV	101,487	44	123	4	0	HQ116624	[95]
	*Cryptophlebia leucotreta* GV	CrleGV	110,907	32	128	3	0	AY229987	[96]
	*Cydia pomonella* GV	CpGV	123,500	45	143	0	1	U53466	[97]
	*Helicoverpa armigera* GV	HearGV	169,794	40	179	9	10	EU255577	[98]
	*Phthorimaea operculella* GV	PhoGV	119,217	35	130	12	1	AF499596	NP
	*Pieris rapae* GV	PrGV	108,592	33	120	0	0	GQ884143	[99]
	*Plutella xylostella* GV	PxGV	100,999	40	120	4	0	AF270937	[100]
	*Pseudaletia unipuncta* GV	PsunGV	176,677	39	183	9	7	EU678671	NP
	*Spodoptera litura* GV	SpltGV	124,121	38	136	0	6	DQ288858	[101]
	*Xestia c-nigrum* GV	XecnGV	178,733	40	181	9	7	AF162221	[15]
*Gammabaculovirus*	*Neodiprion abietis* NPV	NeabNPV	84,264	33	93	5	0	DQ317692	[102]
	*Neodiprion lecontei* NPV	NeleNPV	81,755	33	89	0	0	AY349019	[12]
	*Neodiprion sertifer* NPV	NeseNPV	86,462	33	90	6	0	AY430810	[11]
*Deltabaculovirus*	*Culex nigripalpus* NPV	CuniNPV	108,252	109	50	4	6	AF403738	[56]

1, 2 *hrs* and *bro* refers to homologous regions and baculovirus repeated ORFs, respectively.

3 (TP: this publication; NP: not published, please reference GenBank accession number.)

**Table 2.** OrleNPV genome annotation.

**ORF Name ^1^**		**Position ^2^**			**Intergenic Length Prom**			**Homologues (% aa identity) ^4^**			
		**Left**	**Dir**	**Right**	**(bp)**	**(aa)**	**motifs ^3^**	**EcobNPV**	**ChchNPV**	**OpMNPV**	**AcMNPV**
1	*polyhedrin*	1	>	738	51	246	L	1(95)	1(94)	3(86)	8(89)
2	*orf1629*	789	<	2531	−7	581	?	2(26)	2(28)	-	9(25)
3	*pk1*	2524	>	3327	293	268	L	3(61)	3(49)	1(34)	10(37)
4	*hoar*	3620	<	5245	523	542	E	4(28)	4(23)	-	-
	***rep1***	4273		4348							
5	*orle001*	5768	>	6715	232	316	?	-	-	-	-
	***rep2***	6725		6809							
6	*odv-e56*	6947	>	8080	124	378	L	5(71)	7(39)	146(59)	148(59)
7	*ie-1*	8204	<	10120	70	639	E	6(52)	16(31)	145(30)	147(33)
8	*ep23*	10190	>	10792	118	201	?	7(36)	15(36)	144(38)	146(30)
9	*chtB1*	10910	<	11185	17	92	L	8(64)	14(53)	142(44)	145(52)
10	*odv-e27*	11202	<	12062	89	287	L	9(64)	13(62)	141(47)	144(51)
11	*odv-e18*	12151	<	12420	14	90	L	10(42)	-	-	-
12	*p49*	12434	<	13921	22	496	L	11(57)	11(57)	139(44)	142(47)
13	*ie-0*	13943	<	14794	665	284	L	12(49)	10(39)	138(27)	141(31)
	***rep3***	15148		15218							
14	*me53*	15459	>	16604	90	382	E,L	13(43)	8(34)	137(23)	139(23)
15	*p74*	16694	>	18649	119	652	L	14(71)	17(57)	134(59)	138(58)
16	*iap-3*	18768	<	19052	208	95	E,L	-	-	35(52)	-
17	*dbp-1*	19260	>	20042	28	261	E	27(23)	22(20)	43(28)	25(27)
18	*lef6*	20070	>	20666	253	199	L	16(23)	21(34)	40(35)	28(38)
19	*unknown*	20919	<	21179	212	87	?	17(39)-	20(40)-	39(34)-	29(28)-
20	*p26-a*	21391	>	22224	45	278	L	18(45)	19(44)	132(31)	136(31)
21	*p10*	22269	>	22544	233	92	L	19(83)	18(68)	-	-
22	*cathepsin*	22777	<	23760	2298	328	E,L	51(79)	64(56)	125(64)	127(70)
	***rep4***	25452		25626							
23	*bro-a*	26058	<	26651	345	198	?	-	114(27)	-	-
24	*chitinase*	26996	>	28690	130	565	L	50(72)	65(68)	124(70)	126(69)
25	*unknown*	28820	<	29464	1924	215	?	-	-	-	-
26	*unknown*	31388	<	31858	1052	157	?	-	-	-	-
27	*ctl-1*	32910	<	33068	123	53	E, L	47(62)	74(86)	136(63)	3(41)
28	*dna-ligase*	33191	>	35014	1335	608	?	-	-	-	-
	***rep5***	35314		36297							
29	*gp37*	36349	<	37152	358	268	L	49(57)	67(64)	69(57)	64(58)
30	*bro-b*	37510	<	38265	213	252	?	-	-	-	-
31	*lef9*	38478	<	39959	103	494	?	46(78)	52(74)	65(63)	62(68)
32	*fp25*	40062	>	40685	506	208	E, L	45(70)	51(68)	64(55)	61(54)
33	*chaB2*	41191	>	41502	308	104	?	44(46)	50(48)	63(48)	60(46)
34	*chaB1*	41810	>	42331	72	174	?	43(46)	49(64)	62(49)	59(54)
35	*unknown*	42403	<	42891	365	163	?	42(58)	48(43)	61(35)	57(40)
36	*unknown*	43256	<	43543	−55	96	?	41(38)	47(51)	-	-
37	*unknown*	43488	<	43724	354	79	?	40(46)	-	-	-
38	*vp1054*	44078	<	45112	18	345	E	39(50)	45(51)	58(35)	54(38)
39	*unknown*	45130	>	45396	6	89	?	-	43(42)	-	-
40	*unknown*	45402	>	46421	361	340	?	36(24)	42(31)	-	-
41	*unknown*	46782	<	47198	54	139	?	35(63)	41(55)	56(47)	53(50)
42	*unknown*	47252	>	47863	392	204	?	34(26)	40(32)	-	-
43	*iap-2*	48255	>	48728	43	158	E	54(29)	62(38)	74(29)	71(28)
44	*djbd*	48771	<	49754	−42	328	E,L	33(27)	38(23)	-	-
45	*lef8*	49712	>	52450	−563	913	E	32(72)	37(62)	54(59)	50(63)
46	*unknown*	51887	<	52279	491	131	?	-	-	-	17(60)
47	*unknown*	52770	>	53852	219	361	?	-	-	11(26)	11(25)
48	*unknown*	54071	<	54250	317	60	?	30(46)	35(38)	-	-
49	*bro-c*	54567	>	55595	103	343	?	76(56)	69(45)	-	2(21)
50	*lef12*	55698	<	56429	42	244	?	-	-	46(38)	41(48)
51	*odv-e66a*	56471	<	58537	462	689	?	31(29)	101(39)	50(29)	46(30)
52	*p47*	58999	>	60183	290	395	E	29(63)	33(61)	45(49)	40(52)
53	*dbp-2*	60473	<	61420	386	316	E	15(24)	22(32)	43(24)	25(28)
54	*bv-e31*	61806	>	62489	−66	228	?	26(63)	30(51)	22(57)	38(57)
55	*lef11*	62423	>	62839	−76	139	?	25(58)	29(51)	23(34)	37(37)
56	*39K/pp31*	62763	>	63659	277	299	E	24(51)	28(33)	24(34)	36(34)
57	*unknown*	63936	<	64151	47	72	?	22(45)	-	-	-
58	*ubiquitin*	64195	<	64443	−203	83	L	21(83)	-	-	-
59	*unknown*	64240	>	64479	13	80	?	-	-	-	-
60	*unknown*	64492	>	65079	144	196	?	20(50)	25(31)	26(37)	34(30)
61	*bro-d*	65223	<	66503	419	427	E	89(25)	-	-	2(28)
62	*p26-b*	66922	<	67611	124	230	?	18(28)	19(30)	-	136(27)
63	*iap-2*	67735	<	68691	150	319	E	54(33)	62(32)	-	71(27)
64	*unknown*	68841	<	69230	−1	130	?	55(65)	61(52)	73(41)	68(43)
65	*lef3*	69229	>	70461	278	411	L	56(41)	60(32)	72(28)	67(26)
66	*desmoplakin*	70739	<	73192	−1	818	L	57(40)	59(26)		66(20)
67	*DNA-poly*	73191	>	76406	68	1072	E	58(57)	58(55)	70(41)	65(42)
68	*unknown*	76474	<	76863	7	130	?	59(57)	57(42)	78(26)	-
69	*unknown*	76870	<	77124	114	85	?	60(88)	56(83)	79(40)	76(41)
70	*vlf1*	77238	<	78404	153	389	L	61(84)	76(75)	80(70)	77(74)
71	*unknown*	78557	<	78901	41	115	?	62(38)	-	-	-
72	*gp41*	78942	<	80156	5	405	L	63(68)	78(49)	83(45)	80(55)
73	*unknown*	80161	<	80754	−67	198	?	64(65)	79(69)	84(50)	81(52)
74	*tlp-20*	80687	<	81430	−31	248	L	65(42)	80(47)	-	82(34)
75	*vp91*	81399	>	83870	167	824	L	66(45)	81(42)	86(39)	83(39)
76	*cg30*	84037	<	84801	129	255	?	67(26)	-	89(25)	88(21)
77	*vp39*	84930	<	85961	−1	344	L	68(51)	82(47)	90(46)	89(41)
78	*lef4*	85960	>	87393	162	478	E	69(53)	83(51)	91(42)	90(46)
79	*unknown*	87555	<	87962	196	136	?	70(49)	-	-	-
80	*unknown*	88158	<	88913	−1	252	?	71(57)	84(55)	93(38)	92(42)
81	*unknown*	88912	>	89382	0	157	?	72(69)	85(63)	94(40)	93(46)
82	*odv-e25*	89382	>	90047	152	222	E,L	73(68)	86(64)	95(39)	94(43)
83	*helicase*	90199	<	93912	−43	1238	E,L	74(61)	87(52)	96(35)	95(43)
84	*odv-e28*	93869	>	94384	93	172	?	75(69)	88(65)	97(50)	96(54)
85	*38K*	94477	<	95424	−140	316	L	77(60)	91(55)	99(43)	98(44)
86	*lef5*	95284	>	96198	26	305	?	78(61)	92(59)	100(44)	99(46)
87	*p6.9*	96224	<	96499	68	92	L	79(57)	93(69)	101(62)	100(60)
	***rep6***	96350		96431							
88	*p40*	96567	<	97697	55	377	E,L	80(59)	94(50)	102(41)	101(38)
89	*p12*	97752	<	98105	−4	118	E,L	81(56)	95(34)	103(30)	102(31)
90	*p45*	98101	<	99282	164	394	E, L	82(67)	96(54)	104(39)	103(41)
91	*vp80*	99446	>	101776	107	777	?	83(67)	97(32)	105(27)	104(26)
92	*unknown*	101883	>	102047	41	55	?	84(69)	-	-	-
93	*odv-ec43*	102088	>	103161	38	358	L	85(69)	99(53)	109(46)	109(47)
94	*unknown*	103199	>	103453	527	85	?	86(54)	100(39)	-	-
	***hr1***	103528		103833							
95	*dUTPase*	103980	>	104399	110	140	E	-	119(49)	-	-
96	*unknown*	104509	<	105501	118	331	?	90(40)	-	-	112(45)
97	*endonuclease*	105619	<	106029	216	137	?	88(40)	-	-	-
98	*nrk1*	106245	>	107324	101	360	?	114(42)	106(33)	31(28)	33(30)
99	*unknown*	107425	<	108075	93	217	?	116(64)	107(62)	107(58)	106(55)
100	*bro-e*	108168	<	109211	129	348	?	76(59)	69(47)	-	-
101	*pagr*	109340	<	111118	307	593	?	113(23)	-	-	-
102	*pif3*	111425	<	112030	66	202	?	111(23)	110(50)	115(52)	115(54)
103	*unknown*	112096	<	112998	302	301	?	110(28)	-	-	-
104	*unknown*	113300	<	115057	193	586	?	-	113(27)	-	-
105	*sod*	115250	>	115720	95	157	L	109(61)	115(71)	29(47)	31(75)
106	*ctl-2*	115815	<	115973	311	53	?	47(47)	74(50)	30(50)	3(77)
107	*helicase-2*	116284	<	117591	192	436	?	-	-	-	-
108	*unknown*	117783	>	118103	205	107	?	-	-	-	-
109	*calyx/pep*	118308	<	119270	545	321	L	108(63)	121(50)	129(31)	131(30)
110	*orle002*	119815	>	120387	202	191	?	-	-	-	-
111	*unknown*	120589	>	121710	135	374	?	107(52)	124(44)	113(62)	-
112	*pif2*	121845	<	122999	977	385	L	106(81)	148(68)	20(63)	22(66)
113	*pkip*	123976	<	124533	113	186	L	104(40)	146(29)	-	-
114	*lef2*	124646	<	125275	−43	210	L	103(50)	136(46)	6(38)	6(40)
115	*unknown*	125232	<	125630	232	133	?	-	-	-	-
116	*p24*	125862	>	126572	21	237	L	101(57)	134(55)	127(36)	129(37)
117	*unknown*	126593	<	127081	75	163	?	100(38)	-	-	-
118	*gp16*	127156	>	127458	2813	101	L	99(48)	133(49)	128(36)	130(40)
	***hr2***	127459		130270							
119	*odv-e66b*	130271	>	132241	250	657	E, L	31(41)	101(62)	50(44)	46(30)
120	*rr2*	132491	<	133507	189	339	E	117(76)	122(61)	-	-
121	*unknown*	133696	>	134166	244	157	?	-	-	-	-
122	*unknown*	134410	<	134859	9	150	?	91(76)	126(28)	-	-
123	*unknown*	134868	>	136097	90	410	?	92(34)	125(29)	-	-
124	*alk-exo*	136187	>	137380	1145	398	E	93(48)	127(44)	131(37)	133(39)
125	*fgf*	138525	>	139562	432	346	L	95(36)	130(31)	27(27)	32(30)
126	*pif1*	139994	<	141586	600	531	L	98(66)	131(54)	119(53)	119(54)
	***hr3***	141587		142185							
127	*f-protein*	142186	<	144276	368	697	E,L	118(55)	150(42)	21(23)	23(21)
128	*unknown*	144644	>	147478	308	945	?	119(40)	143(28)	-	-
129	*unknown*	147786	<	148379	241	198	?	121(40)	142(28)	-	-
130	*egt*	148620	<	150149	250	510	E	122(61)	141(63)	14(45)	15(46)
131	*unknown*	150399	<	150758	61	120	?	123(44)	139(49)	-	-
132	*lef1*	150819	>	151508	28	230	E	124(53)	138(50)	13(43)	14(46)
133	*38.7K*	151536	>	152678	104	381	?	125(38)	137(29)	12(24)	13(25)
134	*unknown*	152782	<	153459	182	226	?	-	-	-	-
135	*rr1*	153641	<	155923	257	761	E	126(66)	-	-	-

1 ORFs were named starting from the *polyhedrin* gene (ORF1) to *ribonuclease reductase* 1 (ORF135).

2 Non-coding sequences including direct repeat region and the *hrs* are printed in bold. The direction of each gene is indicated by arrow heads.

3 Promoter motif for each gene is designated as Early (E) or Late (L) based on the consensus elements.

4 Respective homologues for EcobNPV, ChchNPV, OpMNPV, and AcMNPV ORFs are shown followed by the percentage amino acid identities in the brackets.

**Table 3.** OrleNPV unknown and unique ORFs. This table contains some unknown and unique ORFs and their respective genomic positions. ORF 5 (*orle001*) is unique to OrleNPV.

**ORF**	**Name**	**Position**	**Homologue (% aa identity) ^1^**	**Virus**
5	*orle001*	5768–6715	unique	OrleNPV
25	unknown	28820–29464	APNV_p040 (25%)	AnpeNPV
26	unknown	31388–31858	McAVgp043 (31%)	MacoNPV-A
28	*dna ligase*	33191–35014	LyxMNPV_gp020 (32%)	LyxMNPV
59	unknown	64240–64479	APNV-p121 (43%)	AnpeNPV
107	*helicase-2*	116284–117591	Ld-helicase-2 (60%)	LdMNPV
108	unknown	117783–118103	EupsNPV_gp105 (48%)	EupsNPV
110	*orle002*	119815–120387	Agip90 (30%)	AgipMNPV
115	unknown	125232–125630	ORF88_ ApciNPV (41%)	ApciNPV
121	unknown	133696–134166	ORF84_AgseGV (55%)	AgseGV
134	unknown	152782–153459	LdOrf-24_LdMNPV (23%)	LdMNPV

1 Baculovirus homologues with E-value better than the threshold are shown with corresponding amino acid identities in brackets. ORF 110 (*orle002*), a C3HC4 type Zinc finger protein, is the only ORF with E-value worse than threshold. E-values were generated following the previous method [11].
